# Curcumin Nanoemulsion: Characterization and Effect on Cataracts in an In Vivo Animal Model and Ex Vivo Human Model

**DOI:** 10.3390/biom16081166

**Published:** 2026-08-11

**Authors:** Ana G. Castillo-Olmos, Abigail Varela-Pérez, Hugo S. García-Galindo, Joaquín A. Quiroz-Mercado, Kimberly Castañeda-Gutiérrez, Carlos Amero, Enrique Rudiño-Piñera, Mizraim Morales-Mendoza, Cynthia Cano-Sarmiento

**Affiliations:** 1 Unidad de Investigación y Desarrollo en Alimentos, Tecnológico Nacional de México-Instituto Tecnológico de Veracruz, M.A. de Quevedo 2779, Veracruz 91897, Mexico; anagpe_ibq@outlook.com (A.G.C.-O.); abigail_2365@hotmail.com (A.V.-P.); hugo.gg@veracruz.tecnm.mx (H.S.G.-G.); 2 Facultad de Medicina Veterinaria y Zootecnia, Departamento de Medicina Cirugía y Zootecnia para Pequeñas Especies, Universidad Nacional Autónoma de México, Av. Universidad 3000, Ciudad de México 04510, Mexico; jaquiroz@fmvz.unam.mx; 3 LABRMN, Centro de Investigaciones Químicas, Instituto de Investigación en Ciencias Básicas y Aplicadas, Universidad Autónoma del Estado de Morelos, Av. Universidad 2001, Chamilpa, Cuernavaca 62210, Mexico; kimberly.castanedagut@uaem.edu.mx (K.C.-G.); carlosamero@uaem.mx (C.A.); 4 Laboratorio de Bioquímica Estructural, Departamento de Medicina Molecular y Bioprocesos, Instituto de Biotecnología, Universidad Nacional Autónoma de México (UNAM), Av. Universidad 2001, Chamilpa, Cuernavaca 62210, Mexico; enrique.rudino@ibt.unam.mx; 5 Oftalmología, Hospital Regional B de Alta Especialidad ISSSTE Veracruz, Av. Salvador Díaz Mirón, Veracruz 91918, Mexico

**Keywords:** nanoemulsions, curcumin, cataract reversal, human lenses, animal model

## Abstract

Cataracts are the leading cause of reversible blindness worldwide; this condition results from the aggregation of lens proteins. Currently, surgery remains the only treatment; however, there is growing interest in non-surgical approaches, including the use of bioactive compounds incorporated into nanostructured systems designed to enhance solubility, enable controlled release, and improve bioavailability and bioactivity. Among the bioactive compounds investigated, curcumin has attracted considerable attention due to its antioxidant and anti-inflammatory properties, positioning it as a potential anticataractogenic agent. In the present study, curcumin-loaded nanoemulsion was developed via ultrasonication and characterized by average particle size, D90 percentile, ζ potential, and rheological behavior. In addition, its anti-cataract efficacy was evaluated both using an in vivo model in rats and an ex vivo model employing human cataract samples. The resulting curcumin-loaded nanoemulsion exhibited an average particle size of 152 ± 19.79 nm with a monomodal distribution, along with good physical stability over time. The nanoemulsion exhibited apparent viscosity between 30 and 25 mPa·s, at shear rate values (100 to 0 s^−1^), indicating slight shear-thinning behavior. Regarding the effect on cataracts, in the in vivo model, cataract reversal was observed. Furthermore, ex vivo isothermal titration calorimetry (ITC) analyses indicated exothermic heat exchange between the curcumin nanoemulsions and cataract fragments, consistent with binding interactions occurring within lens components, likely involving crystallin proteins. These findings provide biophysical and in vivo evidence that intravitreally administered curcumin-loaded nanoemulsions not only prevent but actively reverse lens opacity, positioning them as a promising non-surgical therapeutic approach for cataract treatment.

## 1. Introduction

It is estimated that around 2.2 billion people worldwide suffer from some degree of visual impairment, with approximately 1 billion of these cases being preventable or yet untreated [1]. Among the leading causes of visual impairment and blindness are cataracts, which affect around 100 million people globally. The World Health Organization (WHO) defines cataracts as the opacity in the lens that scatters light, resulting in a decrease in visual acuity [2]. Cataract is a multifactorial disease and, currently, the process of its development (cataractogenesis) is still under investigation. Several factors, such as aging, smoking, exposure to ultraviolet radiation, frequent alcohol consumption, metabolic and congenital diseases, diabetes, and systemic steroid treatments, contribute to its progress [3,4,5]. These factors cause biochemical and physical alterations in the lens, leading to a state of oxidative stress that triggers imbalances in the detoxification systems located in the lens, lipid peroxidation, osmotic stress, calcium homeostasis, proliferation and induction of apoptosis, as well as nitrosative stress. These phenomena inevitably lead to the aggregation of crystallin proteins: α-, β- and γ-crystallins, resulting in the formation of cataracts [6,7,8,9]. Currently, the only available treatment for this disease is surgery, which involves removal of the crystalline lens and its replacement with an artificial intraocular lens. However, this procedure may be associated with complications, such as post-surgery inflammatory responses, surgically induced astigmatism, posterior capsule rupture or opacification, as well as economic barriers that limit access to treatment. These challenges are particularly pronounced in underdeveloped countries, where access to ophthalmic surgical care is often restricted [5,8,10,11]. For these reasons, it is crucial to identify less invasive, safe, and accessible alternative treatments to prevent or reverse this disease, thereby offering a novel therapeutic approach for this global public health problem.

Several studies have been carried out that evaluate the ability to prevent the formation of cataracts with bioactive compounds, among which curcumin receives particular attention [12,13,14,15,16,17,18]. This compound is the main curcuminoid of the rhizome of *Curcuma longa*, which has been shown to possess beneficial biological activities, such as antitumor, anti-inflammatory, antimicrobial, and antioxidant effects [13,19,20]. The antioxidant activity is attributed to its ability to inhibit lipid peroxidation and neutralize relevant free radicals, since these are involved in cataractogenesis. Studies suggest that curcumin’s antioxidant capacity may help prevent the onset of cataracts; however, to date, there is no evidence demonstrating its ability to reverse established cataracts [13,14,21]. In addition to its antioxidant effects, curcumin has been reported to exert multiple mechanisms of action, including transcriptional regulation of genes such as nuclear factor kappa B (NF-κB) and inducible nitric oxide synthase (iNOS). Through these mechanisms, curcumin may modulate inflammatory responses and regulate the expression of crystallin proteins, thereby preventing their aggregation, a key initiating event in cataract formation [22,23,24,25,26,27]. Despite its therapeutic potential, a major limitation of curcumin as a bioactive compound is its low aqueous solubility and poor bioavailability, underscoring the need to design an appropriate delivery system capable of enhancing its bioactivity. In this context, nanostructured delivery systems, such as nanoemulsions, have emerged as promising strategies to overcome pharmacological challenges associated with ocular administration [28,29]. These systems can enhance the stability, bioavailability, and therapeutic efficacy of lipophilic bioactive compounds, including curcumin, thereby facilitating the development of innovative ophthalmic treatments [30,31,32,33,34,35].

Accordingly, the present study aims to develop and characterize a curcumin-loaded nanoemulsion by evaluating its average particle size, D90 percentile, ζ potential, temporal stability, and rheological properties. Furthermore, the effects of curcumin-loaded nanoemulsions on cataract reversal were assessed using an in vivo model, along with the potential interactions between the nanoemulsified curcumin system and human cataract samples through ex vivo assays employing isothermal titration calorimetry.

## 2. Materials and Methods

### 2.1. Reagents

Curcumin was purchased from LKT Laboratories (St. Louis, MO, USA). Soy phosphatidylcholine (98% purity) was obtained from Avanti Polar Lipids, Inc. (Alabaster, AL, USA). Analytical-grade glycerol (Sigma-Aldrich^®^, St. Louis, MO, USA) was used as a stabilizing agent. Milli-Q^®^ water was employed for nanoemulsion (NE) preparation. The medium-chain triacylglycerides (MCT) blend was acquired from a commercial company (Birdman^®^, Ciudad de Mexico, Mexico); HPLC grade solvents were acquired from (Sigma-Aldrich^®^, St. Louis, MO, USA).

### 2.2. Curcumin Nanoemulsion Formulation

The nanoemulsion (NE) was prepared using the thin-film hydration method described by Ochoa et al. [36], with minor modifications. The ratio between the continuous and dispersed phases was 95:5 [36,37,38]. The dispersed phase consisted of 0.02 g curcumin, 0.5 g medium-chain triacylglycerides (MCT) and 1 g phosphatidylcholine (PC), whereas the continuous phase was composed of 6 g water and 2.5 g glycerol. This formulation resulted in a final curcumin concentration of 0.002 g/mL in the system.

For the preparation of the dispersed phase, 1 g of PC was dissolved in 3 mL of ethanol by magnetic stirring in a Corning PC-420D hot plate (Corning, New York, NY, USA) at 250 rpm for 5 min. Then, 0.5 g MCT were added and stirred for 3 min until complete homogenization. Subsequently, curcumin was incorporated into the mixture and dissolved with agitation at 700 rpm for 15 min. The dispersed phase was subjected to a water bath at 45 °C to remove the ethanol and form a thin lipid film composed of phospholipids, MCTs and curcumin. This film was hydrated with a mixture of 6 g Milli-Q water and 2.5 g glycerol by stirring for 15 min for further processing in the Ultraturrax^®^ T25 rotor-stator homogenizer (IKA^®^, Staufen, Germany) at 20,000 rpm for 3 min thus generating a coarse emulsion. Subsequently, the emulsion was subjected to ultrasonication to reduce the particle size, with a Branson Digital S-450D Sonicator (Branson Ultrasonic Corp., Danbury, CT, USA) with working conditions of 20% amplitude in cycles of 1 min for 3 min, with 50% of the working cycle, resting the sample in an ice bath for cooling.

### 2.3. Characterization of Nanoemulsions

#### 2.3.1. pH Measurement

The pH of the curcumin-loaded nanoemulsion and vehicle was measured using an Ohaus ST2100-F pH meter (Ohaus, Parsippany, NJ, USA). All measurements were performed in triplicate.

#### 2.3.2. Average Particle Size, 90th Percentile (D90) and Zeta (ζ) Potential

The process was carried out with the Zetasizer Nano-ZS90 (Malvern Instruments Inc., Worcestershire, UK). This equipment uses the Dynamic Light Scattering (DLS) technique with a 90° angle at 25 °C to detect the average particle size, particle size distribution and the D90th percentile. Its detection capacity ranges from 0.3 nm to 5 μm. The ζ potential measurement was carried out using Laser-Doppler microelectrophoresis. To prepare the sample for particle size determination, 25 μL of nanoemulsion were diluted in 1 mL of Milli-Q water, and the sample was then deposited into a Versa Fluor Cuvette 170-2415 (Bio-Rad Laboratories, Hercules, CA, USA). For the determination of the ζ potential, the same dilution was used and placed in an electrophoretic cell of the Zetasizer (Nano-ZS90). All measurements were performed in triplicate; the first measurement was made after preparation (T0) and sequentially every 7 days for 28 days (Tf) [39].

#### 2.3.3. Rheology

The NE was rheologically characterized in terms of viscosity and shear stress, by the method of Fuentes et al. [40] with modifications in the geometry and shear rate. Measurements were performed using a MCR 92 rheometer (Anton Paar, Graz, Austria), the geometry used was a concentric double-gap cylinder (DG26.7), equipped with a Peltier temperature control system set at 25 °C. Data acquisition were conducted using Anton Paar RheoCompass software v. 1.30 (Graz, Austria). To obtain a good measurement before each use, motor adjustments were made. The determinations were carried out in duplicate using fresh samples for each measurement.

### 2.4. Sanitization of the Nanoemulsion by UV Treatment

Because the system was designed to be administered ophthalmically, the nanoemulsions must undergo a sanitization process to ensure their safety. The nanoemulsions were sanitized using ultraviolet (UV) radiation. Briefly, 10 mL of the nanoemulsion were transferred into sterile culture plates, and exposed uncovered under the UV light inside a laminar flow hood (Biosafety cabinet, 1300 series A2, Thermo Fisher Scientific, Waltham, MA, USA) for 40 min. Subsequently, the preparation was collected using a sterile syringe and transferred into a sealed glass bottle, where it was stored until used in the animal model. All procedures were performed under sterile conditions [41]. To corroborate that nanoemulsions were not affected by the sanitization process, macroscopic observations and particle size measurements were made before and after sterilization. This UV treatment was not considered a validated terminal sterilization procedure, and sterility and bacterial-endotoxin testing were not performed.

### 2.5. Cataract Induction in an Animal Model

The sodium selenite model was used for cataract induction, as it is an approved and commonly employed method to simulate the formation of nuclear cataracts in humans [19,42,43]. While traditionally performed in neonatal rats via systemic administration, an adapted targeted induction model was implemented using 7-week-old albino Wistar rats (120–140 g) to ensure direct, reproducible oxidative stress to the target tissue.

Animals were allocated to the experimental groups using a completely randomized design. A random allocation sequence was generated, ensuring that each animal had an equal probability of assignment to any group. Randomization was performed before treatment administration (*n* = 3, three eyes). These rats were kept at 23 ± 2 °C and relative humidity between 40 and 70%, with cycles of 12 h light and 12 h of darkness. They were provided with a diet of Purina nutricubes and free access to water. All animal experimental procedures were approved by the Comité de Investigación del Tecnológico Nacional de México (CITEC). Animal care complied with the guidelines of the Association for Research in Vision and Ophthalmology (ARVO) for the use of animals in ophthalmic and vision research, as well as with the Official Mexican Standard NOM-062-ZOO-1999 [44] for the care and use of laboratory animals. The experiment lasted six weeks, from its start to its conclusion.

Cataract induction was performed by an intravitreal injection of 5 μL of sodium selenite (98% purity) 0.01% *w*/*v*. To ensure the integrity of the animals, general anesthesia was administered (tiletamine/zolazepam) at a dose of 40 mg/kg body weight, supplemented with local anesthesia (tetracaine), as well as ocular lubricant (hypromellose) and antibiotic (ciprofloxacin) to prevent infections. Intravitreal injection was performed under the observation of an Olympus stereo microscope model SZ61/SZ51 (Bartlett, IL, USA); 1 mL syringes with 27S gauge needles were used to make a puncture hole at a 45° angle through the sclera into the vitreous cavity, thus releasing intraocular pressure. Then, a Hamilton model 701 RN (Reno, NV, USA) syringe was inserted with a removable 26S gauge needle into the same hole to inject the selenite solution, avoiding contact with the lens. After removing the needle, a drop of antibiotic and anti-inflammatory was applied to each eye for two days [45,46]. Opacity was assessed over a 2-week period when disease was already observed using a Keeler PSL Classic slit lamp (Malvern, PA, USA). The stages of the disease were classified according to Zhao et al. [47] with some modifications: Grade 0: No opacity, Grade 1: A slight degree of opacity, Grade 2: Presence of diffuse opacity involving areas of the lens, Grade 3: Presence of dense opacity involving areas of the lens, Grade 4: Presence of an extensive and dense opacity involving the entire lens.

### 2.6. Evaluation of Cataract Reversal in an Animal Model

The animals were divided according to the treatments administered, forming the following experimental groups: Group I: Healthy Eyes, Group II: Pathological control, Group III: Cataract-induced model treated with curcumin-loaded nanoemulsion. It is worth mentioning that Group IV was evaluated to corroborate that the beneficial effect is due to the bioactive compound and not to the carrier vehicle, so for this group the vehicle was administered to eyes at different disease stages. After cataract formation, treatment was given by two intravitreal injections of 5 μL each, with a waiting time of one week between applications. Both opacity and response to light stimulus were evaluated over a period of 4 weeks using the PSL Classic slit lamp.

The stages of the disease were classified according to Zhao et al. [47] with some modifications: Grade 0: No opacity, Grade 1: A slight degree of opacity, Grade 2: Presence of diffuse opacity involving areas of the lens, Grade 3: Presence of dense opacity involving areas of the lens, Grade 4: Presence of an extensive and dense opacity involving the entire lens. A single-blind evaluation was conducted.

### 2.7. Intraocular Pressure

Intraocular pressure was assessed using an Icare Tonovet TV01 rebound tonometer (Vantaa, Finland) [48]. It was measured at the beginning and end of the cataract reversal study, with measurements performed in triplicate.

### 2.8. Evaluation of the Treatment-Crystallin Protein Interaction Present in Cataract Samples Ex Vivo by Isothermal Titration Calorimetry

Samples from patients with cataracts undergoing phacoemulsification surgery were collected and processed. The samples were centrifuged at 3000× *g* for 10 min at 4 °C to recover the cataract nuclear fragments. Each nuclear fragment was homogenized in balanced saline solution (BSS) until a final cataract fragment concentration of 2 mg/mL was obtained, followed by sonication for 5 min at 40 Hz. Sodium azide (0.005%) was then added as a biocide, and the samples were stored at −20 °C until further use [10]. It is important to mention that this protocol was approved by the Comité de Investigación del Tecnológico Nacional de México (CITEC), as well as by the Comité de enseñanza e investigación del Hospital Regional ISSSTE Veracruz, with protocol number 01-89-224-2022. In addition, written informed consent was obtained from the sample donor in accordance with the principles of the Declaration of Helsinki. Data collection was conducted over the course of one week.

Isothermal titration calorimetry (ITC) experiments were performed at 37 °C on a Malvern ITC200 instrument (Malvern Analytical, Worcestershire, UK). A cataract sample obtained by phacoemulsification (CP), corresponding to an operational protein concentration of 0.05 mM, was loaded into the sample cell, and the syringe was filled with a curcumin nanoemulsion (NE-C) suspension corresponding to an operational concentration of 0.5 mM. Each experiment consisted of 19 injections of 2 μL of NE-C with a 150 s interval between injections and magnetic stirring at 750 rpm [49]. Protein concentration was experimentally estimated from the absorbance measured at 280 nm, considering the dilution factor and a theoretical mean molar extinction coefficient calculated from the nine most abundant crystallin proteins reported in human lens samples. This approach provided an operational estimate of the total crystallin-equivalent concentration used to standardize the calorimetric experiments. Nevertheless, this value does not represent the exact molar concentration of a single protein species or homogeneous binding sites. Likewise, the curcumin nanoemulsion is a multicomponent colloidal system rather than a chemically defined ligand. Therefore, the concentrations reported here were used only to standardize the experiments, and the ITC data were interpreted qualitatively as calorimetric evidence of interactions between two complex systems rather than for quantitative determination of binding parameters.

### 2.9. Statistical Analysis

The results were analyzed using analysis of variance (ANOVA), followed by Tukey’s multiple comparison test. GraphPad Prism version 8.0.1 (GraphPad Software, San Diego, CA, USA), OriginLab version 8.5 (OriginLab Corporation, Northampton, MA, USA), and Duncan’s multiple range test using SPSS software version 31.0.11.0 (IBM Corp., Armonk, NY, USA) were used as appropriate. A significance level of *p* ≤ 0.05 was established.

## 3. Results

### 3.1. Characterization of Curcumin Nanoemulsions

#### 3.1.1. pH Measurement

The pH values of the curcumin-loaded nanoemulsions and vehicle were 4.66 ± 0.01.

#### 3.1.2. Droplet Size Distribution

**Figure 1:**
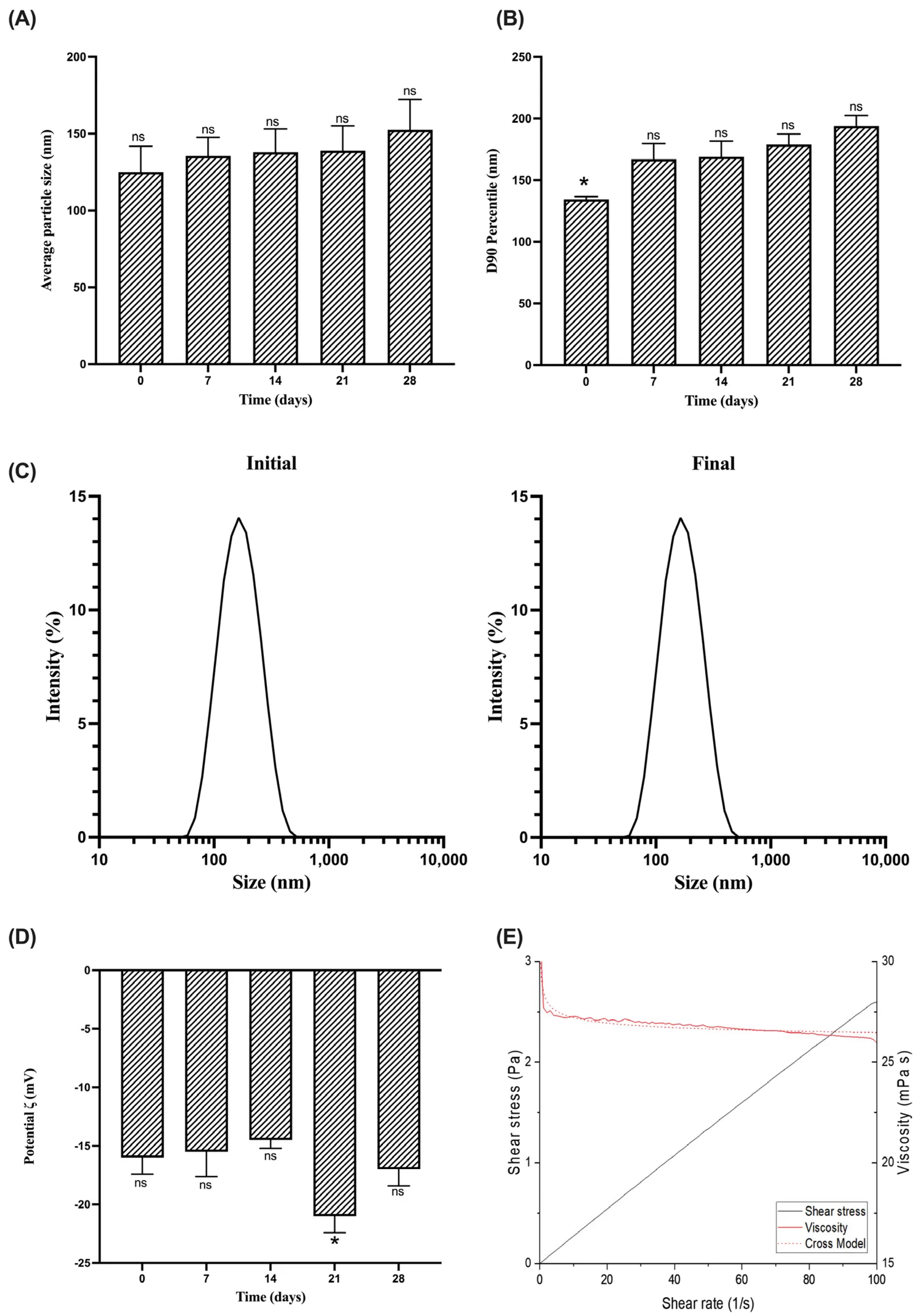
Physicochemical characterization and rheological behavior of the curcumin nanoemulsion: (**A**) Average particle size and (**B**) D90 percentile during storage at 4 °C, (**C**) particle-size distributions at the initial and final storage times; (**D**) ζ potential as a function of storage time at 4 °C; and (**E**) apparent viscosity and shear stress as functions of shear rate at 25 °C. Viscosity data were fitted using the Cross model. Statistical differences over storage time were evaluated by One-way ANOVA followed by Tukey’s multiple comparison test, over time. * *p* < 0.05, ns (not significant).

Initially, the average particle size (APS) of the curcumin nanoemulsion prepared at 20% amplitude was 125 ± 16.87 nm. After 28 days of storage, the droplet size increased to 152 ± 19.79 nm (Figure 1A). However, although the average particle size showed a slight increasing trend during storage, the comparison between day 0 and day 28 revealed no statistically significant differences.

In addition, the D90 value, which represents the upper particle size limit for 90% of the analyzed population, remained within the nanometric range below 300 nm throughout the study (Figure 1B). Moreover, the absence of substantial differences between the mean particle size distribution and the D90 percentile supports the classification of the system as a stable and homogeneous nanoemulsion. It is also important to mention that its initial and final size distribution, had a monomodal shape as can be observed in (Figure 1C).

For the in vivo experiments, a freshly prepared nanoemulsion was subjected to UV treatment immediately before intravitreal administration. This procedure was performed independently of the storage stability study.

After UV treatment of the nanoemulsion, the average size was measured again, which was 153.7 ± 9.10 nm, without a significant change in size after sterilization. No visible color changes or phase separation were observed after UV exposure, indicating the absence of detectable macroscopic instability. However, the chemical stability and residual concentration of curcumin were not directly assessed. Previous studies have reported improved photostability of curcumin following its incorporation into nanoemulsion systems during prolonged UV exposure; nevertheless, these findings cannot be directly extrapolated to the present formulation [50].

#### 3.1.3. Zeta (ζ) Potential

In general, ζ potential values above or below +30 mV and −30 mV are considered indicative of stability against aggregation or coalescence [51,52]. In the experimental system, ζ potential values ranged from −16 to −21 mV, as noted in (Figure 1D).

However, despite showing relatively low ζ potential values for the formulated NE, it showed adequate physical stability, which suggests that the surfactant promotes a stable interface and, together with the stabilizer, an adequate repulsion interaction is achieved for stability between the droplets, which avoids instability phenomena.

#### 3.1.4. Rheology

The viscosity values obtained in the present work at different shear rates ranged from 31.36 ± 1.88 to 25.96 ± 0.81 mPa·s (Figure 1E). According to the viscosity profile obtained in this work, it can be inferred that nanoemulsion will not represent a problem during application. Figure 1E shows two distinct viscosity behaviors. The first occurs at low shear rates (<20 1/s), where a decrease in apparent viscosity of the NE is observed. The second (>20 1/s) was characterized by a gradual decrease in viscosity profile with increasing shear rate. Conversely, the shear stress increased nearly linearly while increasing shear rate (>20 1/s), which may indicate the absence of measurable yield stress and suggests a flow behavior close to that of a Newtonian fluid. Despite this, the nearly linear shear stress and shear rate relationship, fitting the viscosity data to the Cross model (R^2^ = 0.8906), revealed a slight shear thinning behavior in the nanoemulsion.

### 3.2. Induction of Cataracts

**Figure 2:**
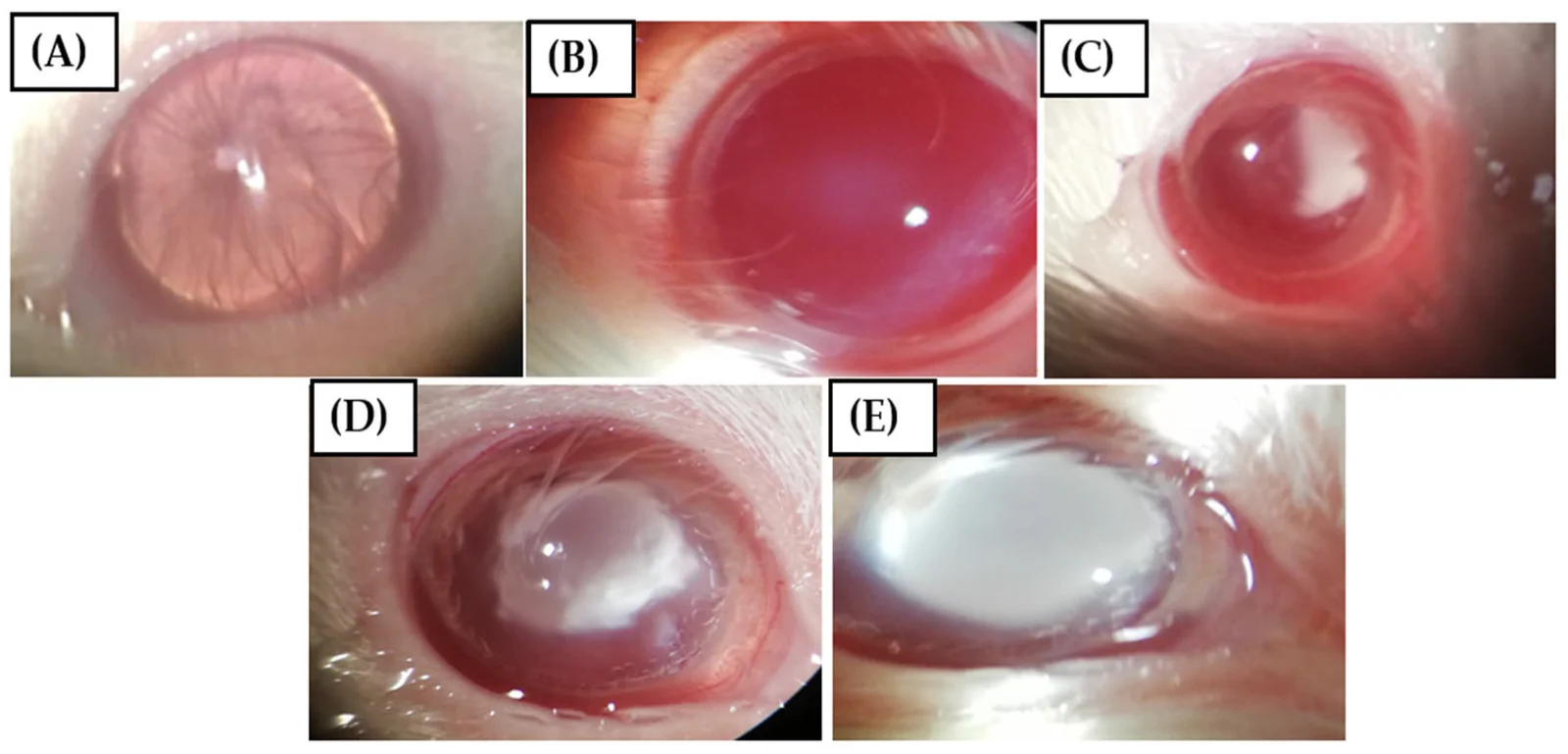
Lens opacification scale of experimental animals (**A**) Stage 0 lens (showing fundus), (**B**) Stage 1 lens, (**C**) Stage 2 lens, (**D**) Stage 3 lens, and (**E**) Stage 4 lens.

The sodium selenite-induced cataract model is one of the most widely used in cataract studies in animals [13,14,18]. In the sodium selenite induction model used in this study, stage 4 of the disease was achieved. Opacification was monitored weekly for two weeks following intravitreal administration of sodium selenite using slit lamp biomicroscopy. Thereafter, lens opacity was classified according to the stages shown in (Figure 2).

### 3.3. In Vivo Model Cataract Reversal

**Figure 3:**
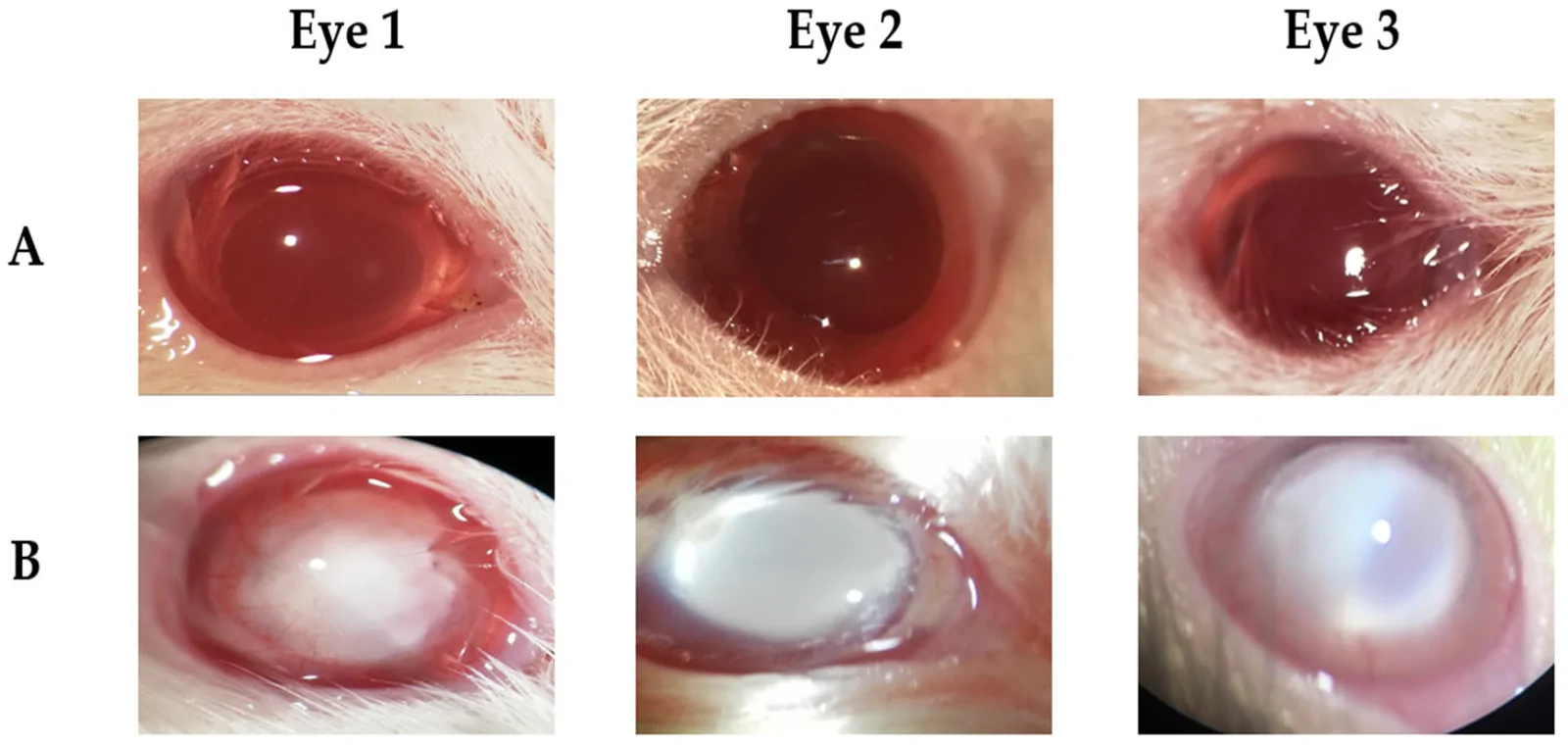
Groups I (**A**), and Group II (**B**) correspond to healthy and pathological controls, respectively.

Although the final evaluation of the experiment was performed at week 4, no apparent changes in lens opacity were observed in the slit-lamp images between weeks 1 and 4 after the second intravitreal injection. Therefore, representative images obtained one week after the second injection are presented in (Figure 3), as they were visually comparable to those acquired at the final evaluation.

The treatments were administered by two intravitreal injections with applications separated by one week. The volume of each injection was 5 μL containing a curcumin dose of (0.01 mg).

**Figure 4:**
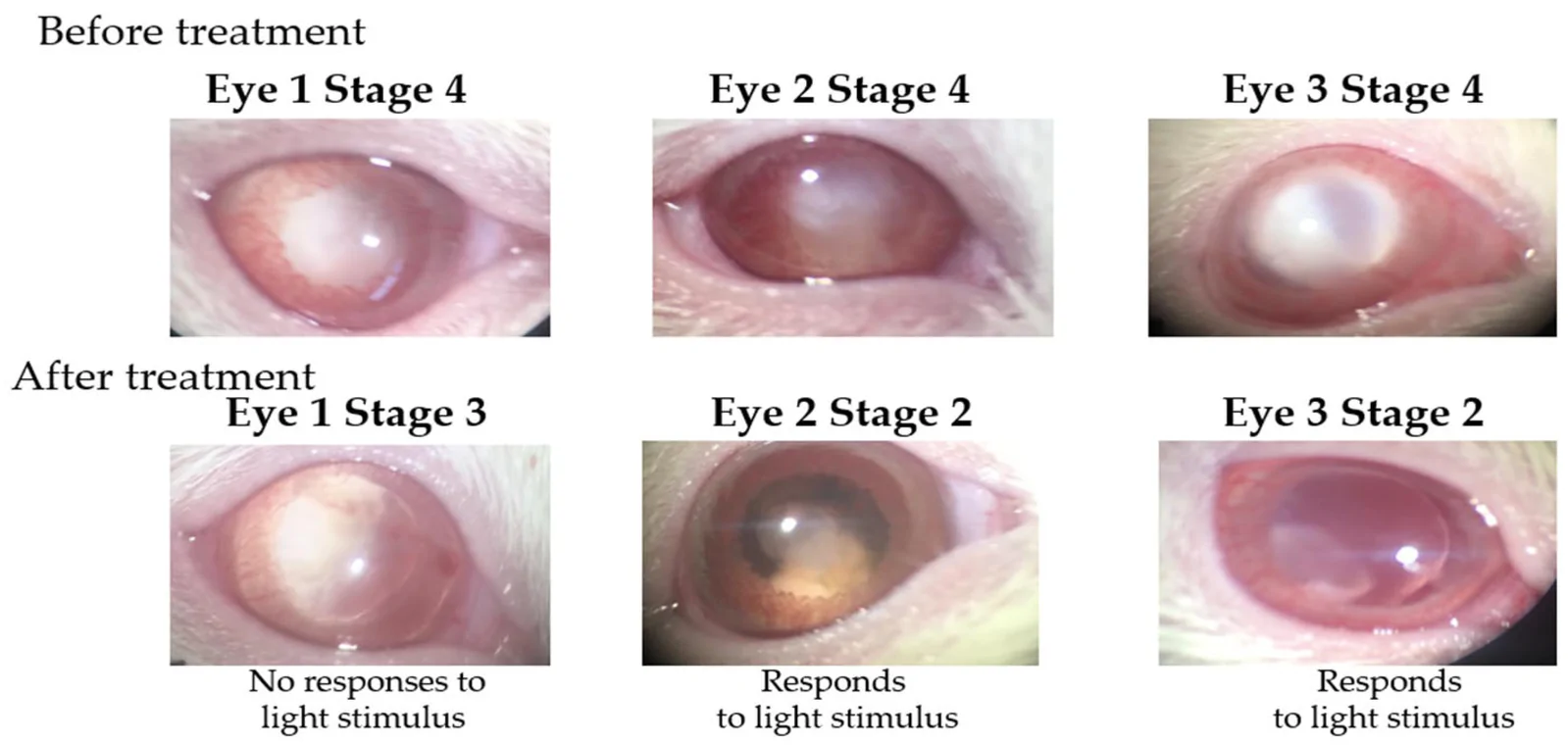
Group III treated with curcumin nanoemulsion before and after treatment.

In Group III treated with curcumin-loaded nanoemulsion, two eyes showed at least two grades in the opacity of the lens, returning to stage 2 cataracts and recovering a positive response to light stimulation. In contrast, one eye showed a reduction of at least one degree in opacity, corresponding to stage 3 cataracts, and did not regain response to light stimulation. These findings are illustrated in (Figure 4).

**Figure 5:**
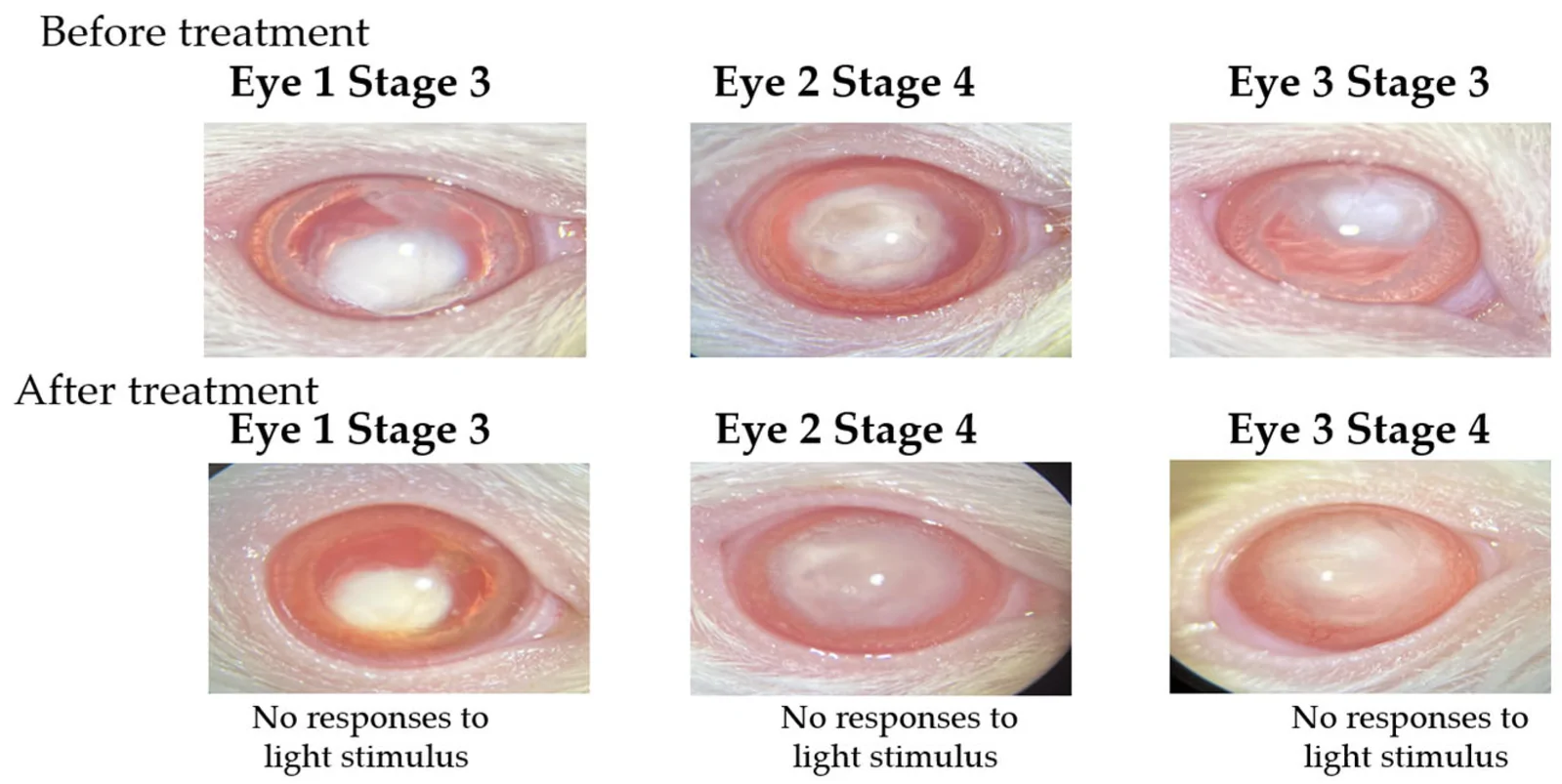
Group IV treated with vehicle before and after treatment.

In Group IV, the eyes treated with the vehicle can be observed where eye 1 remained in stage 3, eye 2 was also maintained in stage 4, while in eye 3 it was even observed that the stage advanced to a grade 4 (Figure 5). In the groups where the curcumin nanoemulsion (Group III) and the vehicle (Group IV) were administered, no changes in behavior or signs of discomfort were observed in the animals compared to the control group (group I) throughout the experimental period.

It is important to mention that during the entire experiment after the intravitreal administration of the treatments, no signs of endophthalmitis, such as secretion, hypopyon, intraocular inflammation or vitreous turbidity, were observed.

### 3.4. Intraocular Pressure

**Figure 6:**
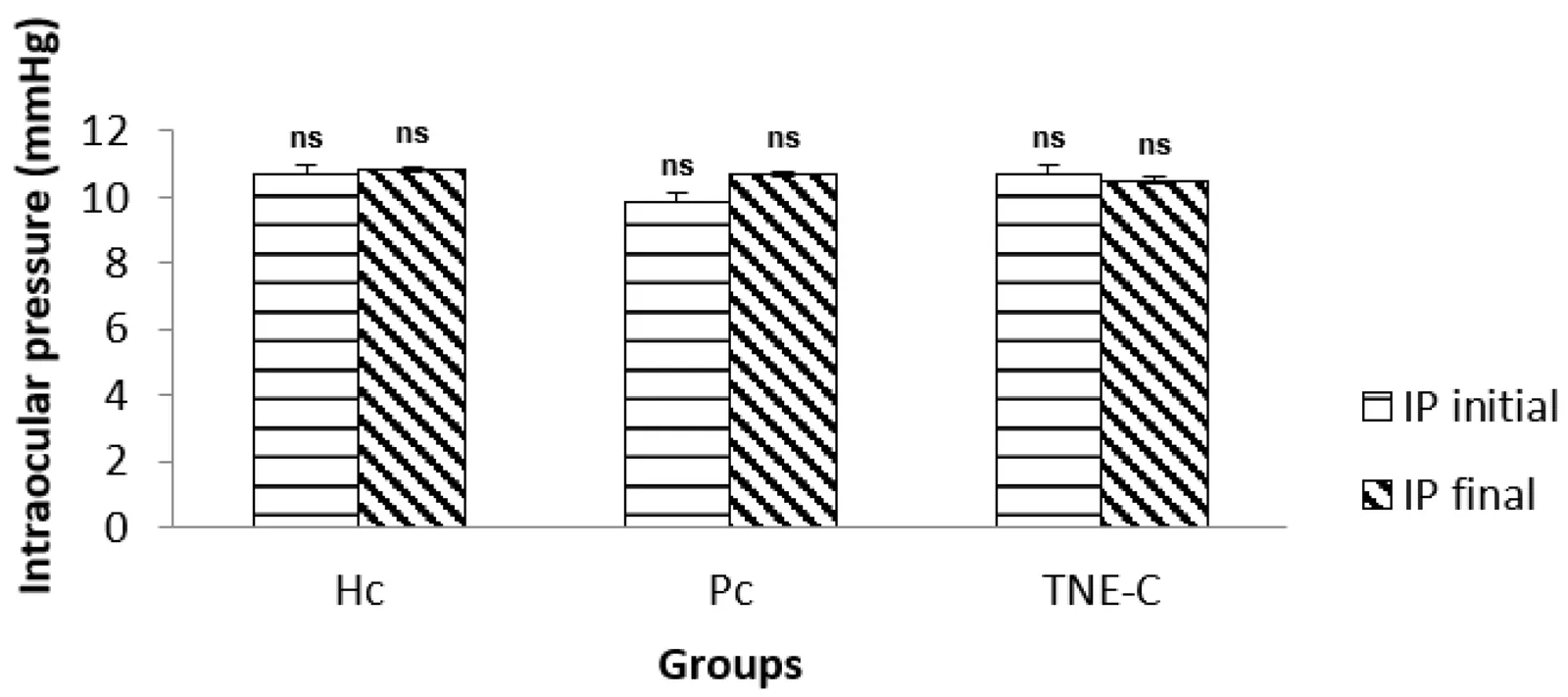
Intraocular pressure (IOP) at the beginning and end of the experiment of all the groups evaluated. Hc (healthy control), Pc (pathological control) and TNE-C (eyes treated with curcumin nanoemulsion). Statistical differences were evaluated by One-way ANOVA followed by Tukey’s multiple comparison test, over time. ns (not significant).

Figure 6 depicts the mean values of intraocular pressure at the beginning and end of week 4 of the experiment, revealing that some groups showed a decrease, while others exhibited an increase in intraocular pressure. However, these changes were not statistically significant and remained within normal values.

### 3.5. Calorimetric Analysis of Curcumin Nanoemulsions Mixed with Human Cataract Fragments Ex Vivo

**Figure 7:**
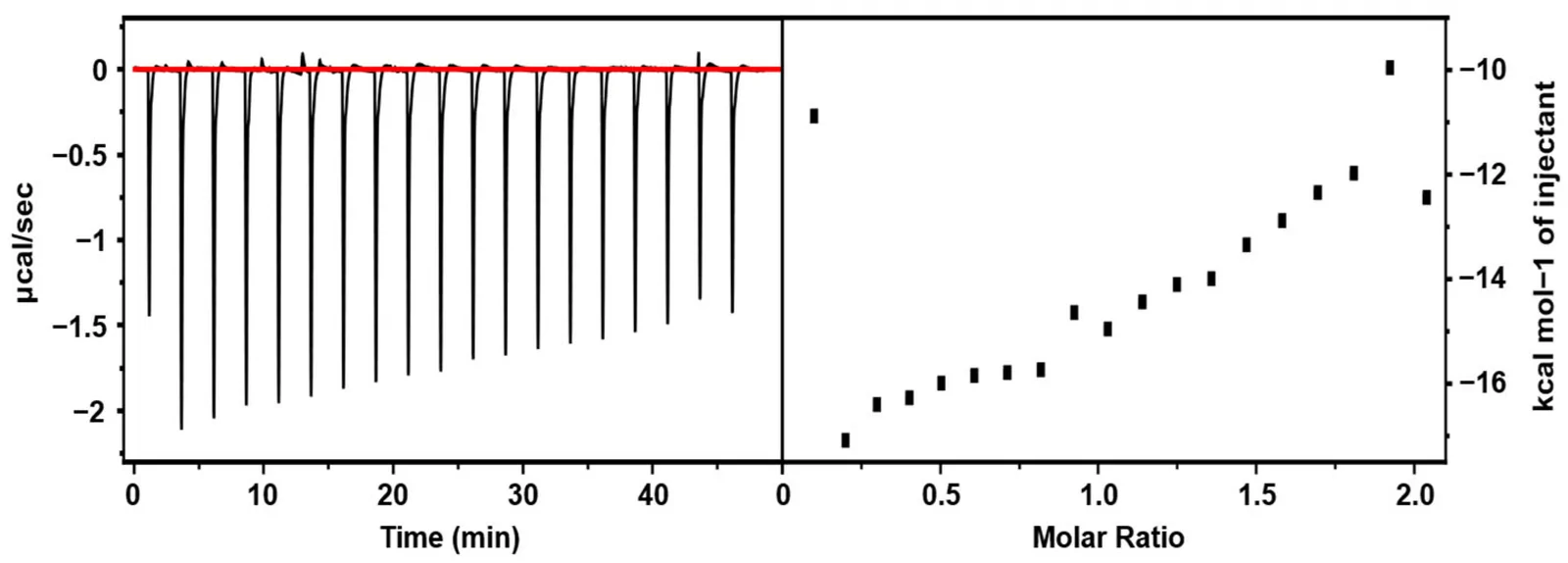
NE-C binding by ITC. Isothermal titration calorimetry of NE-C bound to human cataracts. The left panel shows the experimental titration thermogram, while the right panel shows the heat of reaction.

As an initial approach to investigate the interaction between NE-C and CP, ITC was employed. As shown in (Figure 7), the addition of NE-C produced reproducible exothermic peaks. These thermal events indicate that the addition of nanoemulsion induces measurable energetic changes in the cataract samples, consistent with interactions between the curcumin-loaded nanoemulsion and proteinaceous components present in human cataracts [53]. Because both systems are intrinsically heterogeneous, the ITC experiments were intended only to provide qualitative calorimetric evidence of interaction and were not used to derive quantitative binding parameters.

As the injections progressed, the magnitude of the released heat progressively decreased, suggesting that the major energetic events occurred during the initial additions of the nanoemulsion. A more detailed thermodynamic analysis is not appropriate because of the heterogeneity of both the nanostructured system and the human cataract fragments. The nanoemulsion contains multiple components and potential interaction surfaces, while cataract samples consist of heterogeneous aggregates of lens proteins and other biomolecules [54]. Consequently, the observed calorimetric signal represents the cumulative heat associated with multiple simultaneous processes rather than a single, well-defined molecular binding event. Further studies using purified protein fractions and defined molecular systems will be necessary to identify the specific interactions involved.

## 4. Discussion

The conventional notion that cataracts are irreversible and that surgery is the only solution has been challenged. Currently, there are studies that suggest that certain antioxidants could restore vision by dissolving or disaggregating proteins that lead to clouding in the lens [6,10,16,55]. Studies have documented that oral administration of curcumin exerts a protective effect in cataract models by modulating systemic markers of inflammation and oxidative stress. This has been attributed to free radical sequestration quenching, inhibition of neutrophil and macrophage function, decreased production of pro-inflammatory cytokines, and an increase in the activity of antioxidant enzymes, including SOD, catalase, and glutathione peroxidase [19,20,21,56]. However, because there are physiological barriers that limit ocular bioavailability, the effects of the oral route are usually indirect, reaching the lens through systemic circulation and in lower concentrations. On the other hand, intravitreal or topical administration strategies allow for a more focused action in the intraocular environment. Under these conditions, direct modulation of local inflammatory mediators has been demonstrated, including the reduction in VEGF, IL-6, and MCP-1 in vitreous humor, as well as a decrease in ROS and oxidation-damaged protein levels [57,58]. Other studies report that orally administered curcumin suppresses Hsp70, αA-crystallin, and αB-crystallin expression in rat cataract models induced by streptozotocin or selenite. Therefore, it has been proposed that it could modulate the chaperone activity of α-crystallin proteins [24,56,59,60,61]. This localized effect becomes relevant in pathologies, such as cataracts, where the dysregulation of the redox balance and the inflammatory response favor the aggregation of crystallin proteins. Therefore, based on what has been reported in the literature, our study then focused on the development and characterization of a curcumin-loaden nanoemulsion in terms of its particle size distribution, D90 percentile, ζ potential, and rheology, for subsequent in vivo analysis and ex vivo assays of human cataracts and the emulsified system to evaluate their possible interaction.

The NE formulated was of the oil-in-water (O/W) type, for its formation there must be an adequate emulsification process, as well as a correct selection of the surfactant and its concentration. If there is a high energy input, it can lead to overwork and cause the recoalescence of the droplets, or, at low surfactant concentrations, the size of the droplets would be higher [62,63].

It should be noted that the size, not only of the NE but of any nanostructured system, is crucial, as it will influence the mechanism and ocular delivery of the drug. Because, thanks to their small size and surface characteristics, nanocarriers can cross the eye barriers and deliver therapies directly to the target site. Therefore, although the topical route of administration is the most widely used, its efficacy is limited by the low bioavailability of bioactive compounds, which is caused by the limitations inherent in the eye’s anatomy and the physiological systems responsible for its protection. This led us to the implementation of alternative routes that could favor the release at the target site, particularly the intravitreal route, which allows for a more effective delivery of drugs into the back of the eye [64]. As intravitreal administration was used in this study, it should be noted that the vitreous body is a translucent gel mainly composed of type II, IX, and V/XI collagen fibers, with spaces containing hyaluronic acid. This composition is both a static and a dynamic barrier, where the static barrier refers to the glassy conformation itself, that does not impose many restrictions on molecular mobility. Conversely, the dynamic barrier is related to flow and purification procedures. Also, particle size is crucial in its clearance, since it has a direct impact on the process: both the smaller and lipophilic particles are purified through the posterior segment of the eyeball thanks to their ability to cross the blood–brain barrier (BBB). In contrast, the larger and hydrophilic particles are purified by the anterior segment, through the aqueous humor that comes out. In addition, particle size has an influence on the permeation of the administered treatment. Therefore, it is essential to consider the convective flow from the anterior to the posterior segment of the eye when evaluating its absorption, due to the variation in vitreous pressure. This phenomenon impacts its distribution, especially affecting larger particles [65]. Han et al. [57] described that systems with particle sizes less than 350 nm are ideal in intravitreal administration as they ensure good diffusion in vitreous humor. In this work both the D90 and the average size of the NE droplets were less than 350 nm, that could warrant diffusion and ability to reach the lens, which is the target site to evaluate the effect of cataract reversal. On the other hand, the particle size distribution for the NE prepared where its monomodal behavior is observed with respect to the initial and the final time (28 days). With this trend it can be proposed that the system showed good permanence, ruling out the presence of physical instability phenomena, such as gravitational separation, flocculation, coalescence and Ostwald ripening [66], and that meets the characteristics for a system to be applied intravitreally.

Likewise, it is important to mention that the APS obtained can be compared with the results of Anuchapreeda et al. [67], who reported initial sizes between 56 and 136.5 nm, depending on the type of surfactant and the sonication time used. The same authors noted that the sonication time did not show a significant difference in APS, unlike the surfactant type, obtaining the smaller sizes when using Tween 80. Similarly, Ochoa et al. [36] reported that nanoemulsions with 25 mg curcumin had particle sizes of 108 ± 1 nm at baseline, which increased to 144 ± 3 nm after 30 days; these results were consistent with data from this study. It is important to note that according to Ochoa et al. [36], the sonication amplitude is a crucial factor for particle size, since, although no significant differences in amplitudes between 10 and 30% were observed; however, when amplitude increased between 40 and 80%, the APS increased accordingly. This is because the intensity of the shear force used in the formation of the emulsion depends on the amount of the initial energy, which results from the combination of the applied power and exposure time. Thus, by increasing ultrasound amplitude from 10% to 80%, more energy was supplied to the emulsion, obtaining sizes with the amplitude of 20%, that were similar to that used in the present work. On the other hand, Artiga-Artigas et al. [68], indicated that the concentration of surfactant represents a determining factor in the APS of nanoemulsions. In their study, they evaluate different concentrations of phosphatidylcholine (PC): 2%, 1% and 0.5% in curcumin-loaded nanoemulsions. A concomitant decrease in APS was observed as the concentration of the surfactant increased, obtaining sizes in the range of 322 to 400 nm. These results are consistent with those obtained in this work, in which the use of 10% of PC resulted in sizes of ca. 125 nm. This reduction in APS can be attributed to the ability of low molecular weight surfactants, such as phosphatidylcholine, to rapidly adsorb at the oil–water interface generated during the emulsification process, as noted by Salvia-Trujillo et al. [69]. Therefore, during ultrasonication, the droplets formed by cavitation are rapidly stabilized by surfactant molecules, which reduce interfacial tension and promote more efficient droplet breakdown, thereby facilitating the formation of smaller droplets.

However, although the average particle size at the nanometer scale was confirmed, the dynamic scattering of light does not provide direct information about the particle morphology or internal organization. Therefore, future studies using microscopy and complementary characterization will be necessary to establish the structure of the developed nanoemulsion.

As for the ζ potential obtained, these are relatively low compared to what has been reported in various studies. However, an explanation of the stability of NEs with low ζ potentials is that this parameter provides information about the electrostatic forces of repulsion, without considering the forces of attraction, such as Van der Waals’, and according to the theory of DLVO (Derjaguin, Landau, Verwey, and Overbeek) the stability depends on the sum of both forces of attraction and repulsion. That is why good stability of NEs is possible even with low ζ potential values [70].

As described by Kupikowska-Stobba et al. [71], in general, systems with highly positive or negative ζ potentials involve highly charged particles with a high charge, which provides electrical stabilization that prevents particle aggregation due to electrostatic repulsion. Conversely, systems with low ζ potentials tend to experience coalescence or flocculation phenomena, as attraction outweighs repulsion, leading to physical instability [67]. In terms of surfactant functionality, it has been reported that PC emulsions can be sterically stabilized [72], which explains the stability of the NE obtained in this work. In addition, PC is known to be a lipid with both positive and negative charge (zwitterionic). However, the negatively charged phosphate group is located closer to the aqueous environment, favoring a net negative charge at the surface, resulting in a negative ζ potential [51,68,73].

This characteristic is particularly relevant in the ocular context, as the vitreous humor carries an anionic charge that directly influences the mobility and distribution of administered particles. In this environment, negatively charged particles tend to diffuse freely, whereas positively charged particles may be retained within the vitreous. Therefore, surface charge is a key factor determining the behavior and distribution of particles within the vitreous body [57,65].

The viscosity profile is one of the most important properties in the characterization of these nanostructured systems, as they are designed for intravitreal administration. Their rheological behavior provides insight into their performance after injection in the posterior segment of the eye. Allmendinger et al. [74] reported that solutions with apparent viscosities between 20 and 100 mPa·s, despite requiring greater injection force, do not cause alterations in intraocular pressure (IOP). Based on the apparent viscosity values of curcumin-carrying nanoemulsions, it can be inferred that it will not cause increased IOP when administered to the study subjects, according to the reported range. High viscosities in the formulations may reduce dosing accuracy and require greater injection forces [75]. Such behaviors were reported in Weinmann et al. [76], where they indicate that the application of 30 μL solutions with apparent viscosities of 45 and 80 mPa·s, resulted in errors of up to 40% depending on the syringe used whereas administering 100 μL reduced the error to approximately 10%. This is relevant because of the volume limit allowed to be administered into the vitreous (100 μL), which leads to the need to formulate treatments with higher concentrations, that can lead to an increase in the viscosity of the systems. However, in the present study, the NE with adequate concentration of curcumin was obtained with lower viscosities than those reported by Weinmann et al. [76], thus ensuring minimal error during intravitreal administration.

The viscosity profile showed that the curcumin nanoemulsion exhibits pseudoplastic behavior at low shear rates, evidenced by a decrease in viscosity as the shear rate increases. However, at shear rates above 20 1/s, the viscosity remained practically constant, indicating a tendency toward Newtonian behavior. This result is consistent with the composition of NE, since the rheological properties of nanoemulsions are primarily dominated by the continuous phase. Given that water constitutes approximately 95% of the formulation and the oil volume fraction is 0.05, the rheological response is expected to be dominated by the continuous phase, favoring a nearly constant viscosity at higher shear rates [77].

This rheological behavior is consistent with previous reports on oil-in-water (O/W) emulsions and nanoemulsions. Erramreddy et al. [78] observed that emulsions with a higher aqueous phase content exhibit shear-thinning behavior, characterized by a progressive decrease in viscosity with increasing shear rate. Likewise, Prakash and Parida [79] reported that O/W nanoemulsions initially display non-Newtonian behavior at low shear rates due to the deformation of larger droplets, followed by a Newtonian plateau at higher shear rates where viscosity becomes nearly independent of shear rate. Although the formulations and preparation methods differed from those employed in the present study, the similarity in rheological response suggests that these flow behaviors arise from common microstructural rearrangements occurring in O/W dispersed systems. The reduction in viscosity with increasing shear rate has been attributed to the progressive alignment and rearrangement of dispersed droplets along the flow direction, which decreases hydrodynamic interactions and reduces resistance to flow [79]. The transition toward a Newtonian plateau suggests that droplet–droplet interactions become negligible under these conditions. Such behavior is expected in diluted nanoemulsion, where hydrodynamic interactions dominate over structural rearrangements, allowing the apparent viscosity to approach that of the continuous aqueous phase.

Similarly, Pal and Pal [80] reported that emulsions exhibit Newtonian behavior when the oil volume fraction is less than 0.336. Since the nanoemulsion developed in the present study has an oil volume fraction of 0.05, the predominance of Newtonian behavior at high shear rates is consistent with their observations.

A limitation of the present study is that the osmolality, and isotonicity of the final nanoemulsion were not directly determined. Although the inclusion of a vehicle-treated group allowed nonspecific effects associated with the nanoemulsion matrix and intravitreal administration procedure to be controlled experimentally, these physicochemical parameters are essential for pharmaceutical standardization and future translational development. Therefore, subsequent studies will include direct measurement and adjustment of osmolality, together with sterility, microbial-limit, and bacterial-endotoxin testing. However, something important to mention is that the curcumin-loaded nanoemulsion and vehicle exhibited a pH of (4.66 ± 0.01), which falls within the pH range (4.0–7.4) reported to be well tolerated for intravitreal formulations in both nonclinical and clinical studies according to data reported by Tassew et al. [81]

On the other hand, the UV procedure was applied as a microbial-control treatment based on a previously published methodology. Although no signs of infectious endophthalmitis were observed, this treatment was not considered a validated terminal sterilization method. Therefore, the absence of macroscopic alterations and significant particle-size changes after UV exposure does not confirm the sterility of the formulation, the absence of bacterial endotoxins, or the chemical stability of curcumin. Although previous studies have reported enhanced curcumin photostability following encapsulation in nanoemulsions, this behavior cannot be assumed for the present formulation without direct analytical evidence. Consequently, chromatographic or spectroscopic analyses will be required to determine curcumin concentration and possible degradation products after UV treatment. Likewise, microbial-limit, sterility, and bacterial-endotoxin testing will be necessary in subsequent studies focused on pharmaceutical standardization and clinical translation.

Regarding the classic sodium selenite cataract induction model, although widely used due to its capacity to trigger rapid oxidative and proteolytic changes, we identified significant limitations under our experimental conditions. Initially, the conventional protocol was evaluated; however, systemic administration in low-weight pups often results in highly variable doses at the target tissue, limiting reproducibility. To circumvent this and ensure targeted, reproducible oxidative stress, an adapted intravitreal administration model was implemented. Although this approach differs from the traditional neonatal model, our results show that it effectively generated advanced, reproducible opacities. This allowed for a rigorous assessment of the therapeutic potential of curcumin nanoemulsions on already established cataracts, rather than merely evaluating preventive effects [42].

Faced with this situation, it was necessary to explore alternative strategies that would guarantee adequate exposure of the ocular tissue to the inducing agent. It was decided to use older animals and administer selenite intravitreally, an approach supported by previous studies in other species, particularly in rabbits, where this route has been shown to induce oxidative damage and alterations compatible with cataract formation [44,45]. It is important to note that for ethical and animal welfare reasons, and invasive procedure, the induction was performed only in one eye per individual, which, together with the observed induction efficiency, limited the final number of animals available for therapeutic evaluation. However, this approach allowed us to obtain a suitable model to assess the effect of curcumin nanoemulsion on already established cataracts.

An important thing to mention is that in the present study, the injections were performed by trained personnel using a standardized technique designed to avoid direct contact with the lens. No immediate signs suggestive of injection-related trauma, such as capsular rupture, focal opacity along the needle path, intraocular hemorrhage, or vitreous alteration, were observed during post-injection examination. In addition, lens opacification followed a progressive and reproducible pattern among selenite-treated animals, rather than the immediate or focal presentation usually associated with direct needle injury. Therefore, although a minor contribution of the injection procedure cannot be completely excluded, the absence of clinical signs of mechanical injury and the consistent progression of cataract formation support sodium selenite exposure, rather than needle-induced trauma, as the primary cause of lens opacification.

In addition to overcoming the limitations associated with systemic administration in low-birth-weight animals, the intravitreal route provides a more direct exposure of the lens to the oxidizing agent, favoring the local generation of reactive oxygen species, the imbalance of the antioxidant system and the activation of proteolytic processes related to lens opacification. Therefore, the methodological adaptation implemented preserved the main pathophysiological mechanisms described for the classic selenite model, including oxidative stress, alterations in calcium homeostasis, calpain activation, and crystalline protein aggregation [13,42,82,83].

Therefore, although this approach differs from the traditional neonatal model, the results obtained show that it was able to generate advanced and reproducible opacities, allowing the therapeutic potential of curcumin nanoemulsions to be evaluated. This characteristic represents a relevant difference with respect to most of the previously reported studies, in which treatments are administered before or during the cataract induction process, so its experimental design is mainly oriented towards the prevention or delay of the disease rather than the reversal of an opacity already formed [13,26,55,59,60,61,84,85,86].

Previous research in recent decades has analyzed curcumin as a bioactive compound with the potential to prevent sodium selenite-induced cataracts in various in vivo and in vitro models; however, it has not been evaluated in the reversal of these [13,26,55,59,60,61,84,85,86]. Therefore, in this study, we focused on evaluating curcumin’s ability to achieve a reversal once cataracts have fully formed and stage 4 of the disease has been reached.

In this context, the evaluation of treatments once advanced stages of cataract have been reached allows a more direct assessment of their real therapeutic capacity on damaged tissue. It is also important to consider that the interpretation of preventive studies may be influenced by the efficiency of the induction model itself. Although numerous studies [13,26,55,59,60,61,84,85,86] report a decrease in the incidence or severity of cataracts after the administration of different compounds, in most cases the effective percentage of induction achieved in experimental animals is not reported. Our results demonstrated that induction by sodium selenite does not necessarily achieve 100% efficacy, even when protocols described in the literature are followed, suggesting that a lower occurrence of cataracts could be attributed not only to the protective effect of the treatment evaluated, but also to inherent variations in the efficiency of the induction process.

Therefore, the main strength of the present study lies in the fact that the efficacy of curcumin nanoemulsion was evaluated once the cataracts had been fully established, minimizing the possibility that the observed results were the result of incomplete induction and providing stronger evidence of its potential therapeutic effect on the reversal of lens opacity.

Curcumin may work by boosting antioxidant enzymes, blocking lipid peroxidation, reducing oxidative stress, and affecting lens proteins. This is because one of its mechanisms of action is related to its ability to modulate the response to oxidative stress [87]. This effect is produced, at least in part, by the inhibition of peroxiredoxin 6, a key protein in the antioxidant response of human lens epithelial cells, as demonstrated in an in vitro study by Fatma et al. [88]. Likewise, research in animal models, in which cataracts of varying degrees of severity were induced in rat lenses, showed that the administration of curcumin delayed cataract development by preventing the depletion of antioxidant defenses and limiting the excessive generation of free radicals [19,87].

On the other hand, among the antioxidant systems relevant to lens physiology, glutathione occupies a key role. This tripeptide, made up of glutamic acid (glutamate), glycine and cysteine [87], participates in processes ranging from the detoxification of electrophilic compounds to protection against damage by lipoperoxidation. Numerous studies have assessed that glutathione levels were significantly decreased in models of selenite-induced cataracts [17,55,89]. In this context, Manikandan et al. [13] reported that increased lipid peroxidation in rats with induced cataracts was associated with a negative regulation of glutathione in the lens, an effect that was prevented after the administration of curcumin, which restored its levels and thus confirmed its role as a protective agent against oxidative stress.

Curcumin also exerts effects at the transcriptional level by regulating the expression of genes, such as NF-κB and iNOS, thereby contributing to its anti-inflammatory properties [26]. This gene modulation also plays a role in the regulation of α- and β-crystallins, by prevention of their aggregation—an event considered a key initiating factor in cataract development.

In the same way, an analysis of multiple ranges was carried out using the Duncan test (α = 0.05) for the results obtained, allowing the identification of clear differences between all of the groups evaluated. Although the group with untreated cataract (Group II) presented the highest mean severity (M = 4.00), indicating the absence of reversal and sustained progression of the disease. In contrast, the healthy group (Group I) remained stable throughout the experimental period, remaining in stage 0 (M = 0.00), confirming the validity of the experimental model. On the other hand, the group treated with curcumin-carrying NE showed significantly less opacity compared to the diseased group, although it was still different from the healthy group. However, its intermediate mean (M = 1.67) reflects a shift towards the healthy group, suggesting a partial effect on the reversal of lens opacity. These findings reinforce the therapeutic potential of curcumin-carrying as a pharmacological alternative for the non-invasive treatment of cataracts.

These data confirm the results obtained with curcumin NE where the incorporation of the bioactive compound into a nanocarrier system increased its bioactivity [24,28]. This claim is supported by a previous report by Grama et al. [86], who evaluated the efficacy of nanocurcumin to slow down cataract development in a diabetic model. In the above study the authors found that while free curcumin can delay these cataracts, its clinical use is restricted by its low bioavailability. Therefore, they proposed the administration of nanoparticles loaded with curcumin, showing that an oral administration of 2 mg/day for ten weeks was significantly more effective than curcumin in its free form. Similarly, Yogaraj et al. [90], investigated the effectiveness of curcumin, either encapsulated or in its free form, on the prevention or reversal of sodium selenite-induced cataracts in a Wistar rat offspring model (in vivo), as well as in epithelial cells (HLE-B3) of human infant lens (in vitro). The data showed that the solubility of functionalized nanocurcumin increased 345-fold, and the in vitro release of curcumin was increased approximately 17-fold compared to free curcumin. Nanocurcumin mitigated cataracts in rat pups that received oral doses of 2.5 mg/kg body weight for 24 or 48 h, both before and after selenite treatment. In both cases, this increase in efficacy is attributed to the improved bioavailability and therefore greater oral bioactivity of the compound. An additional analysis that underscores the importance of incorporating compounds with low solubility and bioavailability, such as turmeric-derived compounds, into nanostructured systems is that of Wang et al. [22], who reported that curcumin-loaded nanoformulations offer promising potential for overcoming ocular barriers. This approach opens the door to the clinical application of curcumin-based therapies for the treatment of various ocular diseases, including not only cataracts but also a wide range of other eye conditions.

Likewise, it can be observed that the vehicle failed to improve or reverse cataracts in vivo, so although in a biological environment the vehicles can favor interaction with the target site, the essential therapeutic effect is conferred by bioactive compounds. Research, such as that of Timsina and Mainali [91], has shown that the damage caused by reactive oxygen species induces modifications in the structure of crystalline proteins, resulting in greater light scattering. These alterations in crystalline α facilitate its binding with the membranes of the lens, composed of phospholipids, proteins and cholesterol. Some studies suggest that this interaction is enhanced by ionic forces between α-crystalline and phospholipids, while others attribute the phenomenon to the presence of a hydrophobic surface. In this context, it is plausible that the phospholipids included in the nanoemulsion act as a first step of interaction with the crystalline proteins, which would facilitate the release of the bioactive compounds and, consequently, could explain the observed results.

It is relevant to note that the total amount of curcumin administered in the two intravitreal injections was 0.02 mg per eye. Although this amount was smaller than the lowest dose administered orally in other nanosystems, it is known that higher doses are required when using this route due to the difficulty in getting treatments to reach ocular tissues, mainly for three reasons: first, orally administered treatments must overcome the harsh environment of the gastrointestinal tract and first-pass metabolism. Second, the blood distribution in the eye is considerably smaller compared to the rest of the body, which restricts the accumulation of treatments. In addition, the BBB prevents them from reaching the ocular structures. Similarly, the blood–aqueous barrier (BAB) limits the potency of treatments in the anterior segment of the eye from the general blood flow. Therefore, frequent applications and in high doses is required to achieve the desired therapeutic efficacy, which can generate systemic adverse effects and reduce adherence to treatment [65,89,91]. For this reason, changing the route of administration, as in the case of intravitreal injections, would facilitate the arrival of the treatment at the target site, in addition to reducing systemic absorption and achieving high concentrations in the vitreous chamber. This is because, being a localized route of administration, it facilitates the diffusion of the treatment to the target (the lens), achieving better results with lower concentrations of the bioactive compound. In addition, it is relevant to note that most of the studies reported so far with curcumin have focused on oral administration and with a preventive approach. In contrast, in this work, a therapeutic effect was evidenced, highlighting the reversal of cataracts once they were already formed, which represents a significant advance with respect to the preventive approaches described in the literature.

Intraocular pressure was evaluated to determine whether the intravitreal administration of NE induced pressure changes outside the physiological range observed in rats. Because intraocular pressure may vary according to circadian rhythm, stress, anesthesia, and measurement conditions, values between 9 and 20 mmHg were considered an operational reference range under the experimental conditions. In addition, the animals were examined for signs of intraocular inflammation or uveitis [92,93]. However, all values were within range, and no symptoms of intraocular inflammation were observed.

Isothermal titration calorimetry (ITC) allows the direct measurement of enthalpy changes resulting from molecular interactions. These molecules can be either pure proteins or complex protein systems [48,94]. ITC enables the detection of cumulative thermal events that may reflect conformational changes, which is essential for a better understanding of the molecular nature of these interactions. However, because ITC is highly sensitive to interactions between individual molecules, it does not provide specific information about the type of bonding involved (e.g., hydrogen bonds, hydrophobic interactions) or about more complex interactions involving multiple components [94].

The calorimetric profile obtained in this study suggests that the major energetic events occurred during the initial stages of titration, consistent with the onset of interactions between the treatment and components present in the cataract sample, very likely including lens proteins. This has been proposed by other authors for bioactive compounds with similar properties, such as rosmarinic acid in its free form. This compound not only restored the transparency of human cataracts treated ex vivo but also delayed their formation in vivo. In addition, this bioactive has been shown to bind directly to beta-amyloid and alpha-synuclein aggregates, interfering with oligomer formation in vitro, with a turbidity reduction of up to 92% at ex vivo assay concentrations of 200 μM. In the transmission electron microscopy assays, the authors observed that the size of amorphous cataract particles was reduced to smaller residues. The above is consistent with data of the turbidity assay by using 500 μM–1 mM, in addition to concentrations of up to 40 mM for their in vivo assays, while in this work a concentration of 500 μM was used for the ITC analysis and for the in vivo assays of around 5.43 mM [10].

Based on our results, it is possible to suggest that curcumin contributes to restoring the transparency of the human lens by acting not only on the general ocular environment but also, possibly, through direct interaction with structural components of the lens, such as crystallin proteins, thereby favoring disease reversal. Furthermore, while current evidence indicates that the oral route is useful for modulating the systemic inflammatory and oxidative state—indirectly delaying cataract progression—direct ocular delivery, such as intravitreal administration, offers the distinct advantage of acting locally within the microenvironment of the lens and vitreous humor to significantly enhance the therapeutic effect.

## 5. Conclusions

This study depicts an innovative formulation based on curcumin nanoemulsions, characterized by adequate parameters of particle size distribution, average particle size, D90 percentile and ζ potential that suggest the stability of the system and favor its intravitreal administration. However, additional long-term stability studies, including accelerated stability testing and evaluation under different storage conditions, would further strengthen these findings. Intravitreal application of curcumin nanoemulsions achieved a disease reversal effect in an in vivo model, where low concentrations managed to reverse cataracts in a short period of time. Due to ethical and animal welfare reasons, and invasive procedure, the final number of animals for therapeutic evaluation was limited; however, this approach allowed for the assessment of the effect of a potential cataract treatment.

On the other hand, the ITC experiments showed reproducible exothermic thermal profiles upon addition of the curcumin nanoemulsion to cataract fragments, providing qualitative calorimetric evidence of interactions between both complex systems. Because both the nanoemulsion and the cataract samples are heterogeneous, these experiments were not intended to identify specific molecular binding events or binding-site affinities. Nevertheless, the observed thermal response supports the hypothesis that the nanoemulsion can directly interact with components of the human lens, very likely including crystallin proteins. Therefore, it is possible that curcumin contributes to restoring the transparency of the human lens by acting not only in the ocular environment, as has been reported in the literature, but also, potentially through direct interactions with lens components, such as proteins, thereby contributing to the reversal of the disease.

## Data Availability

The data sets generated during and/or analyzed during the current study are available from the corresponding author on reasonable request.

## Abbreviations

WHO	World Health Organization
NE	Nanoemulsion
MCT	Medium chain triacylglycerides
PC	Phosphatidylcholine
DLS	Dynamic Light Scattering
UV	Ultraviolet
CITEC	Comité de Investigación del Tecnológico Nacional de México
ARVO	Association for Research in Vision and Ophthalmology
BSS	Balanced saline solution
ITC	Isothermal titration calorimetry
CP	Cataract sample obtained by phacoemulsification
NE-C	Curcumin nanoemulsion
APS	Average particle size
D90	Percentile 90
IOP	Intraocular pressure
Hc	Healthy control
Pc	Pathological control
TNE-C	Eyes treated with curcumin nanoemulsion
DLVO	Derjaguin, Landau, Verwey, and Overbeek
ROS	Reactive oxygen species
SOD	Superoxide dismutase
iNOS	Inducible nitric oxide synthase
BBB	Blood-brain barrier
BAB	Blood-aqueous barrier
VEGF	Vascular endothelial growth factor
MCP-1	Monocyte chemoattractant protein-1
NF-κB	Nuclear factor kappa B
IL-6	Interleukin-6
O/W	Oil-in-water
